# Mortality and Case Fatality Due to Visceral Leishmaniasis in Brazil: A Nationwide Analysis of Epidemiology, Trends and Spatial Patterns

**DOI:** 10.1371/journal.pone.0093770

**Published:** 2014-04-03

**Authors:** Francisco Rogerlândio Martins-Melo, Mauricélia da Silveira Lima, Alberto Novaes Ramos, Carlos Henrique Alencar, Jorg Heukelbach

**Affiliations:** 1 Department of Community Health, School of Medicine, Federal University of Ceará, Fortaleza, Brazil; 2 Anton Breinl Centre for Public Health and Tropical Medicine, School of Public Health, Tropical Medicine and Rehabilitation Sciences, James Cook University, Townsville, Australia; Royal Tropical Institute, Netherlands

## Abstract

**Background:**

Visceral leishmaniasis (VL) is a significant public health problem in Brazil and several regions of the world. This study investigated the magnitude, temporal trends and spatial distribution of mortality related to VL in Brazil.

**Methods:**

We performed a study based on secondary data obtained from the Brazilian Mortality Information System. We included all deaths in Brazil from 2000 to 2011, in which VL was recorded as cause of death. We present epidemiological characteristics, trend analysis of mortality and case fatality rates by joinpoint regression models, and spatial analysis using municipalities as geographical units of analysis.

**Results:**

In the study period, 12,491,280 deaths were recorded in Brazil. VL was mentioned in 3,322 (0.03%) deaths. Average annual age-adjusted mortality rate was 0.15 deaths per 100,000 inhabitants and case fatality rate 8.1%. Highest mortality rates were observed in males (0.19 deaths/100,000 inhabitants), <1 year-olds (1.03 deaths/100,000 inhabitants) and residents in Northeast region (0.30 deaths/100,000 inhabitants). Highest case fatality rates were observed in males (8.8%), ≥70 year-olds (43.8%) and residents in South region (17.7%). Mortality and case fatality rates showed a significant increase in Brazil over the period, with different patterns between regions: increasing mortality rates in the North (Annual Percent Change – APC: 9.4%; 95% confidence interval – CI: 5.3 to 13.6), and Southeast (APC: 8.1%; 95% CI: 2.6 to 13.9); and increasing case fatality rates in the Northeast (APC: 4.0%; 95% CI: 0.8 to 7.4). Spatial analysis identified a major cluster of high mortality encompassing a wide geographic range in North and Northeast Brazil.

**Conclusions:**

Despite ongoing control strategies, mortality related to VL in Brazil is increasing. Mortality and case fatality vary considerably between regions, and surveillance and control measures should be prioritized in high-risk clusters. Early diagnosis and treatment are fundamental strategies for reducing case fatality of VL in Brazil.

## Introduction

Visceral leishmaniasis (VL) or Kala-azar is a Neglected Tropical Disease (NTD) and continues being a significant public health problem in several regions of the world [1]. There are 400,000–500,000 new cases annually worldwide, with 40,000–50,000 deaths [1]–[3]. VL is endemic in 65 countries (distributed in Asia, Europe, Middle East, Africa and the Americas), and 90% of cases are reported from Bangladesh, India, Brazil, Nepal, Ethiopia and Sudan [1]. In Latin America, VL is caused by *Leishmania infantum* (*Leishmania chagasi*) [4], [5]. The vectors are sandflies of genus *Lutzomyia* (*L. longipalpis* as main species) [4]. The domestic dog (*Canis familiaris*) is the main reservoir of the parasite in urban areas [4]–[6]. VL is present in 12 Latin American countries, with 90% of all cases reported in Brazil [7], [8].

In Brazil, there have been important changes in disease transmission patterns [9], [10]. Originally, VL was characterized as an endemic disease of rural areas and focal occurrence, mainly in the Northeast region of the country [9], [11]. Since the 1980s, the geographical distribution of VL has expanded with increasing urbanization, and the disease has spread to all regions of the country, with continuing expansion to non-endemic areas [6], [10]–[13]. Currently, the disease is distributed in 21 of the 27 Brazilian states, with about 3,500 annual cases [10].

VL is a potentially fatal disease if not diagnosed and treated precociously [3], [6], [10], [14], [15]. Despite the wide geographical distribution and high case fatality of VL in Brazil, there are only a limited number of systematic population-based studies investigating the dynamics of mortality related to VL [14]–[16]. The Visceral Leishmaniasis Control and Surveillance Program (VLCSP) in Brazil is based on reducing the morbidity and case fatality through the early diagnosis and treatment of human cases, vector control, elimination of reservoirs, and health education [4], [10]. Thus, knowledge of the magnitude of mortality and case fatality by VL in Brazil is essential to subsidize planning processes, monitoring and evaluation of the impact of interventions and the effectiveness of measures to VL control. In this study, we analyzed the magnitude, temporal trends and spatial distribution of mortality related to VL in Brazil, from 2000 to 2011.

## Methods

### Study Area

**Figure 1:**
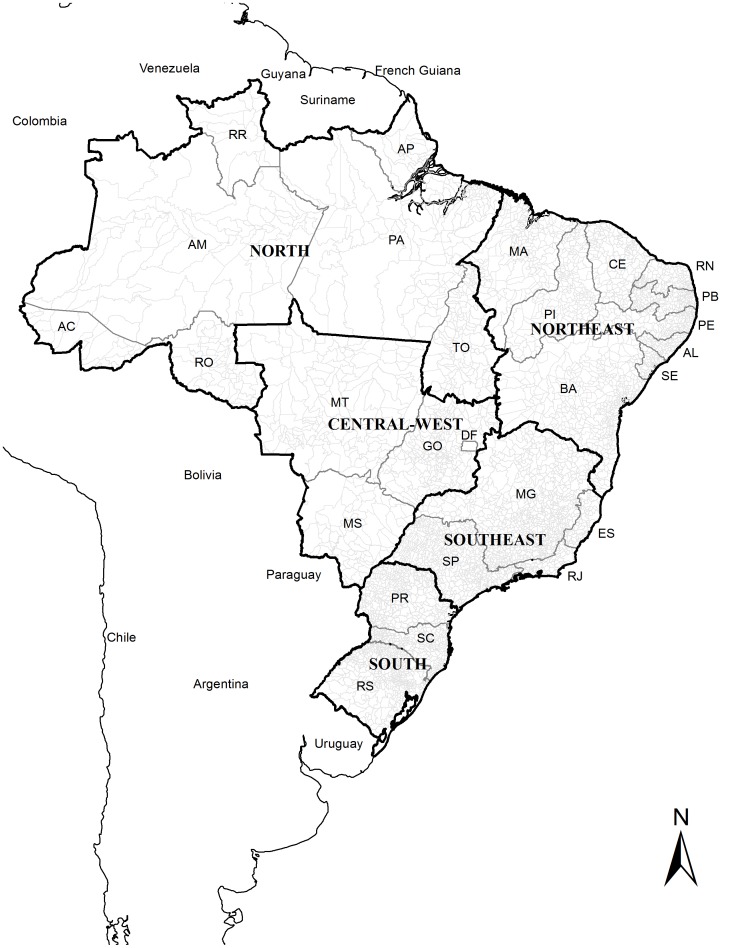
Brazil with its regions and 27 Federative Units.

Brazil, located in South America, has a total territory of 8.5 million km^2^ and an estimated population of 201 million (2013). It is divided into five geographic regions (South, Southeast, Central-West, North and Northeast), 27 Federative Units (26 states and one Federal District) and 5,570 municipalities (Figure 1) (*Instituto Brasileiro de Geografia e Estatística – IBGE*; http://www.ibge.gov.br).

### Study Design and Population

We performed a study of time series and spatial analysis, based on secondary data of deaths related to VL in Brazil, from 2000 to 2011 (mortality database compiled and available until 2011). We analyzed epidemiological characteristics, temporal trends and spatial patterns of mortality related to VL in Brazil, with identification of high-risk areas.

### Data Sources

Mortality data were obtained from the Mortality Information System (*Sistema de Informação sobre Mortalidade - SIM*) of the Brazilian Ministry of Health. *SIM* data are based on death certificates, consisting of standardized forms to be filled out by the physicians in charge. Death certificates contain demographic data (sex, age, education, race, marital status, date of death, place of residence and occurrence of death) and clinical information (causes of death). *SIM* data are public domain and freely available at the website of the Informatics Department of the Unified Health System (*Departamento de Informática do Sistema Único de Saúde - DATASUS*, http://tabnet.datasus.gov.br/cgi/deftohtm.exe?sim/cnv/obt10uf.def). We processed a total of 324 mortality data sets, with about 12.5 million entries. The process of downloading data sets and data processing of *SIM* database has been described in detail previously [17], [18].

We included all deaths in Brazil between 2000 and 2011, in which VL was mentioned at any position on the death certificate, either as underlying or as associated cause of death (multiple causes of death). VL as a cause of death corresponded to subcategory B55.0 (included within the category Leishmaniasis – B55) of the Tenth Revision of the International Statistical Classification of Diseases and Related Health Problems (ICD-10) [19].

Population data were obtained from the Brazilian Institute of Geography and Statistics (*Instituto Brasileiro de Geografia e Estatística – IBGE*, http://tabnet.datasus.gov.br/cgi/deftohtm.exe?ibge/cnv/popuf.def), based on the National Population Census (2000 and 2010) and population estimates for inter-census years (2001–2009 and 2011). VL cases data were obtained from the nationwide Reportable Disease Information System (*Sistema de Informação de Agravos de Notificação* – *SINAN*, http://dtr2004.saude.gov.br/sinanweb/) and epidemiological reports of the Brazilian Ministry of Health [20].

### Statistical Analysis

We described available characteristics of the study population: sex, age, region and state of residence, race/color, marital status, residence and occurrence in state capital, educational level and causes of death (underlying or associated). Descriptive statistics included the calculation of mean and standard deviation (SD) for continuous variables and of absolute numbers and proportions (with their respective 95% confidence interval [95% CI]) for categorical variables.

Crude and age-standardized mortality rates stratified by sex, age group and place of residence (macro-regions, states and municipalities) were calculated by dividing the number of deaths in each calendar year by the population, expressed per 100,000. Age-adjusted mortality rates were calculated by direct method, using the 2010 Brazilian population as standard. Age categories employed in calculation of age-specific mortality rates were: <1 years, 0 to 14 years, 15 to 29 years, 30 to 39 years, 40 to 49 years, 50 to 59 years, 60 to 69 years and ≥70 years. We estimated case fatality rates by region of residence (2000–2011), sex and age group (2001–2011), by dividing the number of deaths due to VL by the total number of notified VL cases, multiplied by 100.

Analysis of time trends was performed using joinpoint regression model over the 12-year period [21]. Time trends were calculated using age-standardized rates (sex and Brazil’s five geographic regions), age-specific rates and case fatality rates (Brazil’s five geographic regions) as the dependent variables and the year of occurrence as the independent variable. The objective of this analysis was to identify a significant change in the linear slope of the trend (on a log-scale) during the study period [21]. The best fitting points (the joinpoints) were chosen at which the rate changes significantly (increase or decrease). The analysis started with the minimum number of joinpoints (e.g., 0 joinpoints; which a straight line) and tested whether one or more joinpoints (in our analysis up to 3) are significant and must be added to the model. Each significant joinpoint that indicated a change in the slope (if any) was retained in the final model. To describe linear trends by period, the estimated annual percent change (APC) was computed for each of these trends by fitting a regression line to the natural logarithm of the rates using calendar year as a regressor variable. To simplify the trend comparison for these indicators where more than one slope was identified, we also calculated the average annual percent change (AAPC) over the entire period (when available), based on an underlying joinpoint model. This was estimated as the geometric-weighted average of the APC, with the weights equal to the length of each time interval segment [21], [22]. An increase in indicators was considered to have occurred when the trend was toward growth and the minimum value of the confidence interval was bigger than 0 (zero). Conversely, a reduction was considered to have occurred when there was a decline in the trend and the maximum value of the confidence interval below 0 (zero). Regardless of the trends, stability was defined when the confidence interval included zero.

Lastly, we analyzed the spatial distribution patterns of mortality related to VL in Brazil using municipalities of residence (n = 5,565; territorial division of 2010) as the geographical units of analysis. Spatial analysis methods and Geographic Information System (GIS) tools were used to evaluate the geographic distribution and spatial dependence of mortality rates related to VL in Brazil. Two strategies were applied as a basis for the construction of spatial distribution maps of deaths related to VL. To correct random fluctuations and provide greater stability in mortality rates mainly in small municipalities and small populations, first, crude mortality rates were estimated as four-year means (2000–2003, 2004–2007 and 2008–2011), and for the total period (2000–2011). Then, the mortality rates were adjusted (smoothed rates) by the Local Empirical Bayesian method [23].

After descriptive spatial analysis, we evaluated the presence of global spatial dependence using Global Moran’s I index on smoothed mortality rates [24]. Moran’s I index ranges from −1 to +1: values close to zero indicate spatial randomness; positive values indicate positive spatial autocorrelation; and negative values indicate negative spatial autocorrelation [24]. Then, we evaluated the existence of local autocorrelation (Local Index of Spatial Association – LISA) by means of Local Moran’s index [25]. To identify critical or transition areas, we used the Moran Scatterplot Map, based on Local Moran’s Index, to compare the value of each the municipality with neighboring municipalities, and to display spatial dependence, as well as the identification of spatial patterns [25]. The quadrants generated in this technique are interpreted as follows: Q1 – High/High (positive values, positive means) and Q2 – Low/Low (negative values, negative means), indicating points of positive spatial association or similar to neighbours, i.e., representing municipalities with high and low mortality rates surrounded by municipalities with high and low mortality rates, respectively; Q3 – High/Low (positive values, negative means) and Q4 – Low/High (negative values, negative means), indicating points of negative spatial association, i.e., municipalities with low and high mortality rates surrounded by municipalities with high and low mortality rates, respectively. The first two categories represent concordance areas and the last two transition areas [25]. For spatial representation of the Moran Scatterplot Map, Moran Maps were used considering municipalities with statistically significant differences (p<0.05). High risk areas (hot-spots) for mortality caused by VL were considered when formed by municipalities covered by class Q1 (High/High) of the Moran Map.

Data analysis was performed using Stata software version 11.2 (StataCorp LP, College Station, TX, USA). Joinpoint regression analyses were carried out using Joinpoint Regression Program version 4.0.4 (US National Cancer Institute, Bethesda, MD, USA). ArcGIS software version 9.3 (Environmental Systems Research Institute, Redlands, CA, USA), and TerraView software version 4.2 (*Instituto Nacional de Pesquisas Espaciais, INPE, São José dos Campos, SP,* Brazil) were used for input, processing, analysis and presentation of cartographic data, calculation of global and local spatial autocorrelation indicators, and construction of thematic maps.

### Ethics Statement

This study is based on secondary data, and all presented information is public domain. No variables allowed identification of individuals. Thus, approval of the study by an Ethical Review Board was not necessary.

## Results

Between 2000 and 2011, a total of 12,491,280 deaths were recorded in Brazil. VL was mentioned in 3,322 (0.03%) of these, with 2,727 (82.1%) as an underlying cause and 595 (17.9%) as an associated cause. The average number of deaths related to VL was 277 per year, ranging from 198 in 2001 to 350 in 2009. In the same period, 41,015 new VL cases were notified (average annual incidence rate of 1.84 cases/100.000 inhabitants). Average annual age-adjusted mortality rate in the period was 0.15 deaths per 100,000 inhabitants (95% CI: 0.13–0.16) and case fatality rate 8.1% (95% CI: 7.8–8.4). The relative increase of indicators after inclusion of associated causes of death was 25% (0.15 vs. 0.12 deaths/100,000 inhabitants as underlying cause) and 22.7% (8.1% vs. 6.6% as underlying cause), respectively.

### Epidemiological Characteristics

Predominating characteristics were: male gender (63.4%), brown color/race (61.9%), age <15 years (37.0%; mean: 30.3; median: 29.1; SD ±26.0) and residents in the Northeast region (56.0%) (Table 1). From all states in Brazil, Minas Gerais had the largest proportion of cases (15.3%). Despite the majority residing in municipalities of the rural hinterland (78.5%), the place of occurrence of deaths was mostly in hospitals (95.4%) and in Brazilian state capitals (59.7%) (Table 1).

**Table 1:** Epidemiologic characteristics of deaths related to VL in Brazil, 2000–2011 (n = 3,322).

Characteristic	n	%	95% CI
**Sex**			
Male	2,107	63.4	61.8–65.1
Female	1,215	36.6	34.9–38.2
**Age group (years)** a			
<1	434	13,1	12.0–14.3
1–14	793	23.9	22.5–25.4
15–29	460	13.9	12.7–15.1
30–39	402	12.1	11.0–13.3
40–49	369	11.1	10.1–12.3
50–59	327	9.9	8.9–10.9
60–69	233	7.0	6.2–8.0
≥70	294	8.9	7.9–9.9
**Race/color** a			
Brown/Mixed	1,794	61.9	59.4–63.2
White/Caucasian	706	24.4	23.2–26.6
Black/Afro-descendant	350	12.1	10.8–13.4
Indigenous	30	1.0	0.7–1.5
Yellow/Asian-descendant	19	0.7	0.4–1.1
**Education level (years)** a			
None	581	33.5	31.3–35.8
1–3	484	27.9	25.8–30.1
4–7	436	25.1	23.1–27.2
8–11	180	10.4	8.9–11.9
≥12	53	3.1	2.3–.4.0
**Marital status** a			
Single	1,349	58.8	56.8–60.1
Married	692	30.2	28.3–32.1
Widow	156	6.8	5.8–7.9
Divorced/Separated	72	3.1	2.5–3.9
Stable union	24	1.0	0.7–1.6
**Region of residence**			
Northeast	1,860	56.0	54.3–57.7
Southeast	703	21.2	19.8–22.6
North	419	12.6	11.5–13.8
Central-West	332	10.0	9.0–11.1
South	08	0.2	0.1–0.5
**Federal Unit/State of residence (region)**			
Minas Gerais (Southeast)	507	15.3	14.1–16.5
Maranhão (Northeast)	466	14.0	12.9–15.2
Ceará (Northeast)	417	12.5	11.4–13.7
Bahia (Northeast)	339	10.2	9.2–11.3
Piauí (Northeast)	244	7.3	6.5–8.3
Mato Grosso do Sul (Central-West)	202	6.1	5.3–6.9
Tocantins (North)	202	6.1	5.3–6.9
Pará (North)	199	6.0	5.2–6.9
São Paulo (Southeast)	185	5.6	4.8–6.4
Pernambuco (Northeast)	154	4.6	3.9–5.4
Others states^b^	407	12.3	11.2–13.4
**Place of occurrence** a			
Hospital	3,145	95.4	94.7–96.1
Residence	111	3.4	2.8–4.0
Other healthcare facilities	20	0.6	0.4–0.9
Public areas	19	0.5	0.3–0.9
**Residence in state capital**			
Yes	715	21.5	20.1–23.0
No	2,607	78.5	77.0–79.9
**Occurrence in state capital**			
Yes	1,984	59.7	58.0–61.4
No	1,338	40.3	38.6–42.0

a Data not available in all cases (age group: 10, race/color: 423, education level: 1,588, marital status: 1,029, and place of occurrence: 9).

b Rondônia, Amazonas, Roraima, Amapá, Rio Grande do Norte, Paraíba, Alagoas, Sergipe, Espírito Santo, Rio de Janeiro, Paraná, Rio Grande do Sul, Mato Grosso, Goiás, and Distrito Federal.

95% CI: 95% confidence intervals.

The highest average annual mortality rates were observed in males (0.19 deaths/100,000 males) and residents in the Northeast (0.30 deaths/100,000 inhabitants) region, while the highest case fatality rates were found in males (8.8%) and residents in the South (17.8%) region (Table 2). The states of Tocantins (1.28 deaths/100,000 inhabitants), Mato Grosso do Sul (0.74 deaths/100,000 inhabitants) and Piauí (0.67 deaths/100,000 inhabitants) had the highest average annual mortality rates, while the states of Amazonas (54.5%), Rondônia (28.6%) and Rio Grande do Sul (28.6%) had the highest case fatality rates. Highest age-specific mortality rates are found in the extreme age groups, especially in <1 year-olds (1.03 deaths/100,000 inhabitants) and ≥70 year-olds (0.36 deaths/100,000 inhabitants). Highest case fatality rates were found in the older age groups, mainly in ≥70 year-olds (43.8%) and 60–69 year-olds (23.3%) (Table 2).

**Table 2:** Average annual number of deaths and cases, age-adjusted and age-specific mortality rates (per 100.000 inhabitants), and case fatality rates related to VL in Brazil, 2000–2011.

Variables	VL deaths(average annual/range)^a^	VL cases (averageannual/range)^a^^,^ b	Mortality rate (per 100,000 inhabitants)^c^^,^ d	Incidence rate (per 100,000 inhabitants)^b^^,^ c	Case fatality rate (%)^b^
**Sex**					
Male	176 (116–225)	2,012 (1,485–2,344)	0.19	2.19	8.82
Female	101 (78–125)	1,273 (956–1,469)	0.10	1.34	7.92
**Age group (years)**					
<1	36 (26–45)	325 (192–407)	1.03	9.16	9.47
1–14	66 (46–91)	1,617 (1,271–1,905)	0.13	3.12	3.95
15–39	72 (50–95)	826 (654–1,008	0.07	1.02	8.64
40–59	58 (26–91)	370 (178–582)	0.15	1.06	16.46
60–69	19 (8–31)	87 (44–140)	0.22	0.98	23.28
≥70	24 (12–45)	58 (26–114)	0.36	0.85	43.79
**Region of residence**					
North	35 (15–52)	595 (299–866)	0.24	4.00	5.87
Northeast	155 (134–167)	1,960 (1,463–4,029)	0.30	3.82	7.91
Southeast	59 (21–98)	601 (240–782)	0.08	0.76	9.75
South	1 (0–2)	4 (0–10)	<0.01	0.01	17.77
Central-West	28 (15–40)	258 (123–354)	0.26	1.96	10.40
Brazil	277 (198–350)	3,418 (2,448–4,858)	0.15	1.84	8.10

a Average annual number of deaths and cases. Range: Annual variation in the number of deaths and cases - minimum and maximum value in the period.

b VL cases data for sex and age available for period 2001–2011.

c Average annual incidence or mortality rates, calculated using the average number of cases or deaths due to VL as a numerator and population size in the middle of period as a denominator.

d Age-standardized (Brazilian Census 2010 population) and age-specific mortality rates.

### Temporal Trends of Mortality Indicators

**Figure 2:**
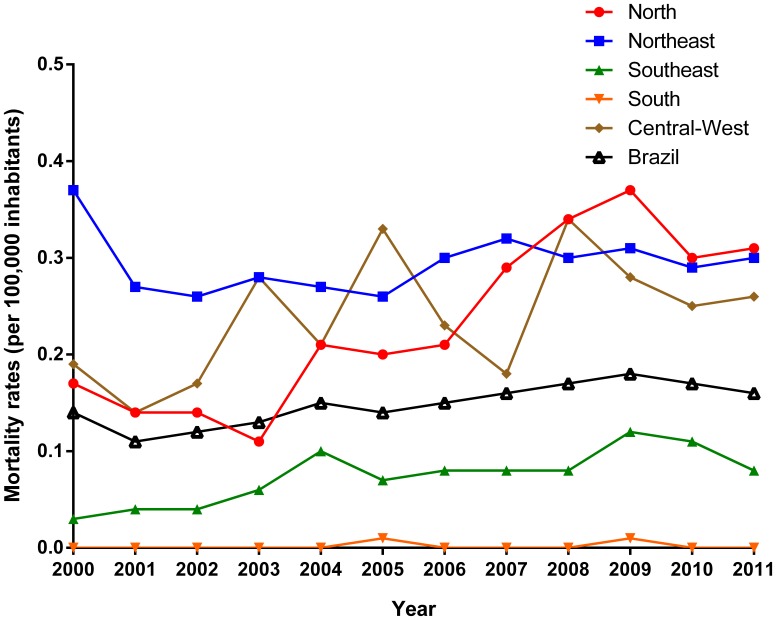
Trends of age-adjusted mortality rates (per 100,000 inhabitants) related to VL in Brazil and regions, 2000–2011.

Age-adjusted mortality rates presented significant increasing trend at national level (APC: 3.3%; 95% CI: 1.7 to 5.0) over the entire period, with different patterns between regions (Figure 2; Table 3). There was a significant increase of mortality rates in the North (APC: 9.4%; 95% CI: 5.3 to 13.6) and Southeast (APC: 8.1%; 95% CI: 2.6 to 13.9) regions. The mortality rates in Northeast region decreased not significantly during 2000–2002 (APC: −14.3%; 95% CI: −28.6 to 2.9), with posterior significant increase during 2002–2011 (APC: 2.0%; 95% CI: 0.3 to 3.7). The mortality rates in the Central-West region remained stable over time (APC: 3.8%; 95% CI: −0.7 to 8.6) (Figure 2; Table 3). Both males (APC: 3.9%; 95% CI: 2.0 to 5.9) and females (APC: 2.3%; 95% CI: 0.4 to 4.3) showed significant increase mortality over the period (Table 3). Age-specific mortality rates ranged among age groups, with significant increase trends in the age group of 40–50 years (APC: 8.0%; 95% CI: 2.6 to 13.7), 50–59 years (APC: 7.2%; 95% CI: 3.3 to 11.2) and ≥70 years (APC: 9.2%; 95% CI: 5.7 to 12.9). The other age groups remained stable (Table 3).

**Table 3:** Joinpoint regression analysis of mortality indicators related to VL in Brazil, 2000–2011.

Indicator/Variable	Trend 1			Trend 2			Trend 3			Entire period	
	Period	APC	95% CI	Period	APC	95% CI	Period	APC	95% CI	AAPC	95% CI
**Mortality rates**											
***Sex***											
Male	2000–2011	3.9*	2.0 to 5.9							3.9*	2.0 to 5.9
Female	2000–2011	2.3*	0.4 to 4.3							2.3*	0.4 to 4.3
***Age group (years)***											
<1	2000–2011	0.3	−3.1 to 3.9							0.3	−3.1 to 3.9
1–14	2000–2011	−1.7	−4.7 to 1.4							−1.7	−4.7 to 1.4
15–29	2000–2002	−17.9	−41.4 to 17.1	2002–2009	5.8	−0.3 to 12.3	2009–2011	−13.0	−39.0 to 24.1	−2.3	−8.8 to 4.7
30–39	2000–2011	3.2	−0.4 to 6.9							3.2	−0.4 to 6.9
40–49	2000–2011	8.0*	2.6 to 13.7							8.0*	2.6 to 13.7
50–59	2000–2011	7.2*	3.3 to 11.2							7.2*	3.3 to 11.2
60–69	2000–2011	5.2	−0.1 to 10.7							5.2	−0.1 to 10.7
≥70	2000–2011	9.2*	5.7 to 12.9							9.2*	5.7 to 12.9
***Region of residence***											
Brazil	2000–2011	3.3*	1.7 to 5.0							3.3*	1.7 to 5.0
North	2000–2011	9.4*	5.3 to 13.6							9.4*	5.3 to 13.6
Northeast	2000–2002	−14.3	−28.6 to 2.9	2002–2011	2.0*	0.3 to 3.7				−1.2	−4.1 to 1.8
Southeast	2000–2011	8.1*	2.6 to 13.9							8.1*	2.6 to 13.9
South	2000–2011	NC								NC	
Central-West	2000–2011	3.8	−0.7 to 8.6							3.8	−0.7 to 8.6
**Case fatality rate**											
***Region of residence***											
Brazil	2000–2011	3.4*	1.0 to 5.8							3.4*	1.0 to 5.8
North	2000–2011	1.9	−1.2 to 5.1							1.9	−1.2 to 5.1
Northeast	2000–2011	4.2*	0.7 to 7.8							4.2*	0.7 to 7.8
Southeast	2000–2011	3.5	−0.1 to 7.3							3.5	−0.1 to 7.3
South	2000–2011	NC								NC	
Central-West	2000–2011	−0.3	−4.7 to 4.3							−0.3	−4.7 to 4.3

APC: annual percent change; AAPC: average annual percent change; 95% CI: 95% confidence intervals.

NC: Not calculated - Estimated APC analyses could not be performed when an observation contained a zero mortality or case fatality rate.

*Significantly different from 0 (p<0.05).

**Figure 3:**
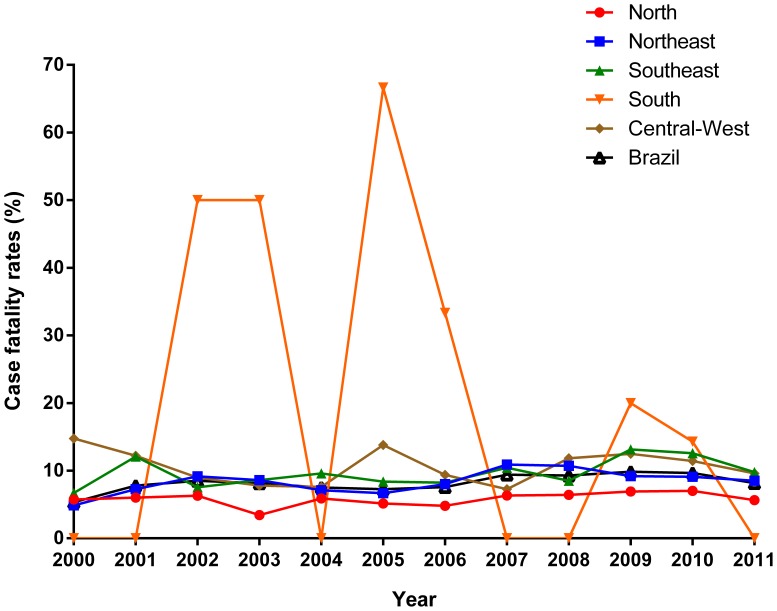
Trends of case fatality rates related to VL in Brazil and regions, 2000–2011.

Similar to mortality rates, case fatality rates in Brazil increased significantly (APC: 3.4%; 95% CI: 1.0 to 5.8) over time (Figure 3; Table 3). The Northeast (APC: 4.2%; 95% CI: 0.7–7.8) region presented significant increase, while the North (APC: 1.9%; 95% CI: −1.2 to 5.1), Southeast (APC: 3.5%; 95% CI: −0.1 to 7.3) and Central-West (APC: −0.1%; 95% CI: −4.7 to 4.3) regions remained stable (Figure 3; Table 3).

### Spatial Patterns of Mortality Related to VL

**Figure 4:**
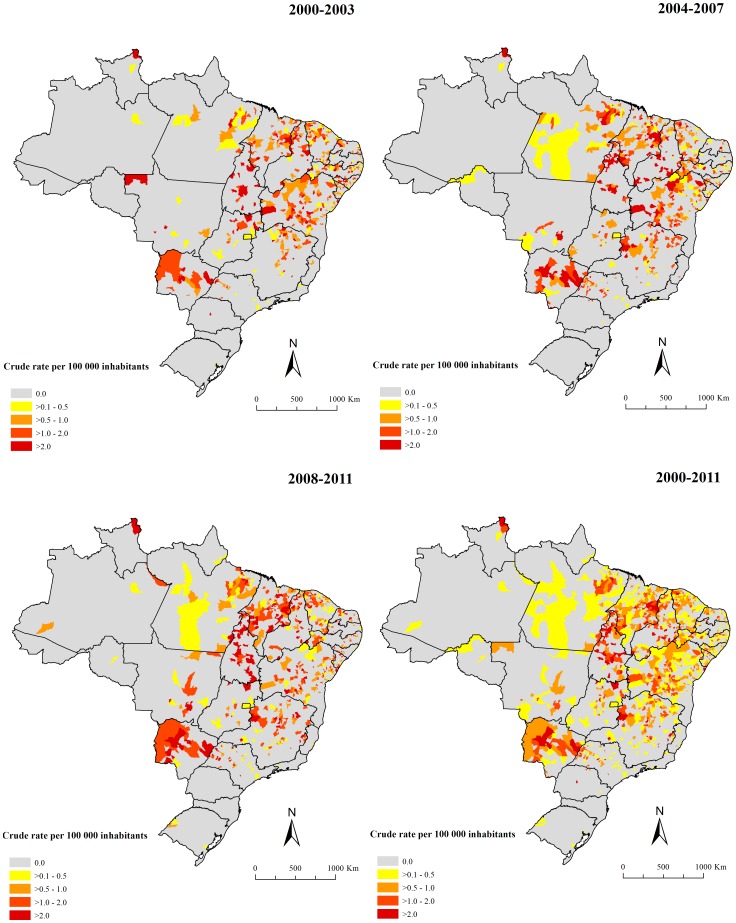
Spatial distribution of average annual mortality rates related to VL (per 100,000 inhabitants) based on multiple causes of death by municipalities of residence, Brazil, 2000–2011.

**Figure 5:**
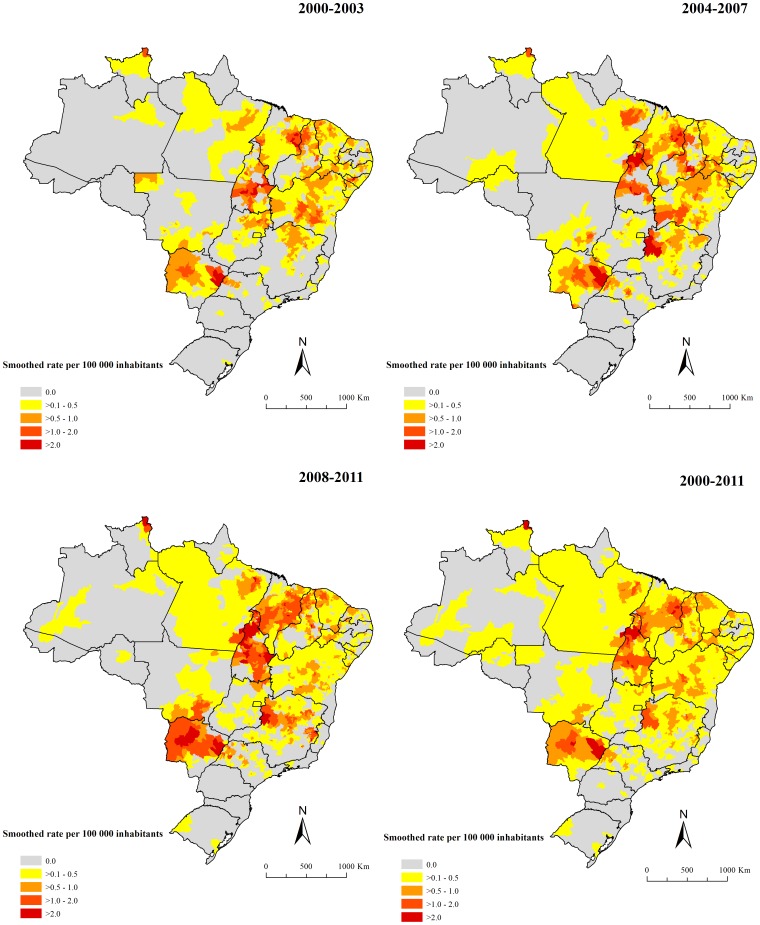
Spatial distribution of average annual mortality rates (per 100,000 inhabitants) related to VL after smoothing by Bayesian Local Empirical method by municipalities of residence, Brazil, 2000–2011.

In the period, 18.1% (1,010/5,565) of the Brazilian municipalities in 25 of 27 states reported at least one death related to VL. Figures 4 and 5 present the spatial distribution of the average annual crude and smoothed mortality rates, respectably. The Bayesian method generated more stable corrected mortality rates (Figure 5). Average annual crude rate reached a maximum of 9.5 deaths per 100,000 inhabitants, while the maximum of the smoothed indicator was 3.6 deaths per 100,000 inhabitants. In general, the thematic maps show the presence of municipalities and/or clusters of municipalities with high mortality rates related to VL (>0.5 deaths per 100,000 inhabitants) in states of North, Northeast, Southeast and Central-West regions. The largest concentration was found in the Northeast, covering areas in all nine states of this region (Figures 4–5). There were also areas with high mortality rates encompassing most of Tocantins and Mato Grosso do Sul states, central-north and west regions of Minas Gerais state, west region of São Paulo state, east and north regions of Goiás state and northeast region of Pará and Roraima states (Figures 4–5).

**Figure 6:**
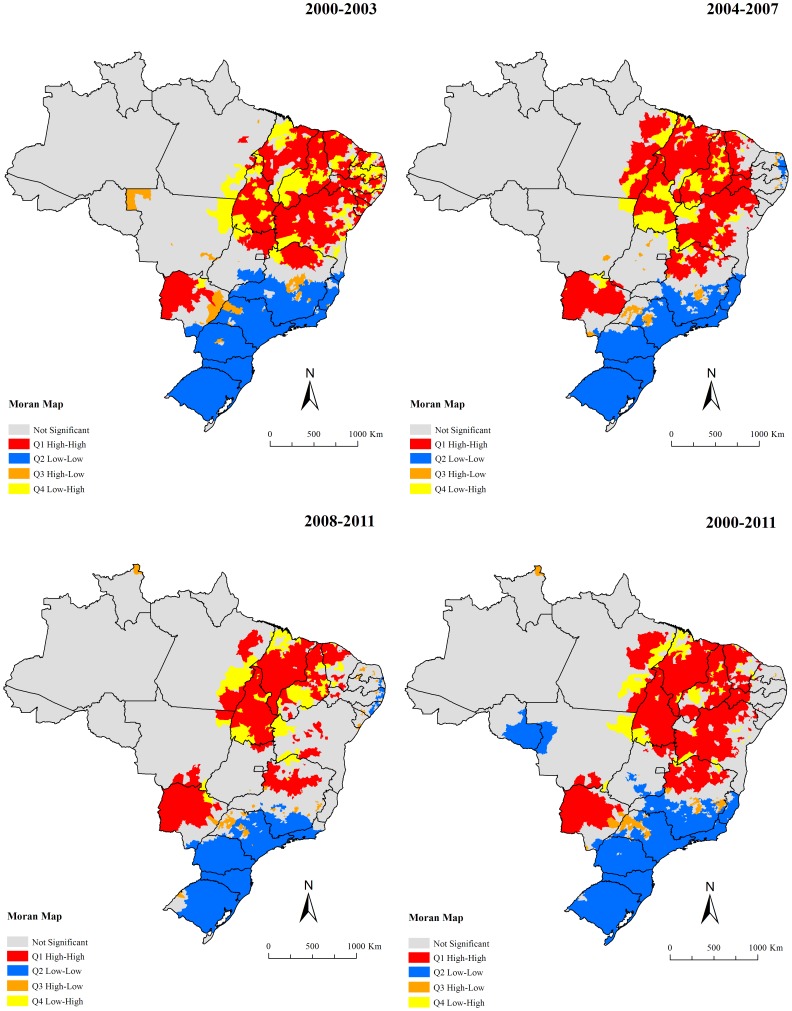
Moran maps of average annual mortality rates related to VL by municipalities of residence, Brazil, 2000–2011.

Global Moran’s I index for the sub-periods and entire period showed significant positive values ranging from 0.17–0.25 (p<0.01), evidencing the existence of spatial dependence among mortality rates of the municipalities with similar patterns. Figure 6 presents the clusters of municipalities, identified according to Local Moran’s index for smoothed mortality rates and visualized through Moran Map. A major cluster of municipalities with high mortality rates (High/High) was identified, encompassing a geographic range covering east of Pará state, most of the states of the Northeast region, Tocantins state, north of Goiás state and northwest of Minas Gerais state (Figure 6). There was another high risk clusters covering almost the entire of Mato Grosso do Sul state and the south region of Mato Grosso state (Figure 6). Clusters of municipalities with low mortality rates (Low/Low) were located encompassing almost the entire South region, in large part of Southeast region, well as areas with isolated municipalities in the Central-West and North regions (Figure 6).

## Discussion

The present national population-based study provides a comprehensive overview of mortality related to VL in Brazil. Mortality indicators presented an increase at national level, with different patterns among regions, sex and age groups. Higher mortality rates were concentrated in the youngest and eldest (<1 and ≥70 year-olds) and residents in endemic areas. We further identified high mortality risk clusters. The use of multiple causes of death increased mortality indicators considerably, evidencing underestimation of deaths in most mortality studies using underlying causes of death as a basis for calculation [18], [26]–[28].

The highest relative frequency of deaths related to VL was observed in male children, confirming the pattern of higher occurrence of VL in this population in Brazil [6]. The higher male susceptibility is still a matter of debate, and may be caused by socioeconomic, environmental and behavioral factors [16], [29]. In Brazil, the highest number of cases and deaths reported in child populations [6], [10], [29], [30] can be explained by more frequent contact with reservoir animals and vectors as compared to adults, higher rates of nutritional deficiency, and an immune status in formation, leading to reduced specific immunity [29], [30]. Highest case fatality rates were observed in advanced age and was highest in subjects >70 years of age. This draws attention to the frequent comorbidities such as cardiovascular diseases in this age group, which increase the risk of death due to VL [14]–[16], [31], [32]. Furthermore, the predominance of mortality in colored people may confirm the strong social nature of VL and its status of an important NTD in Brazil [33].

The Northeast region (main endemic region) presented the highest mortality rates and risk of death of approximately twice of the national average. The high number of cases and deaths in this region reflects environmental and social vulnerability favoring spread of the disease [6]. However, the sharp increase of the number of cases and deaths in other Brazilian regions reflects geographic expansion and urbanization process in recent decades [6], [13]. Factors related to changes in the occurrence of geographic patterns as result of intense migration of rural populations to the periphery of medium and large cities, poor, precarious housing, uncontrolled deforestation and an increasing number of infected dogs contributed to the expansion of the VL and increased case fatality rates [13], [14], [34].

On the other hand, the South region, which is considered a non-endemic area, showed the highest case fatality rates. As absolute numbers were low in this region, the rates differed from one year to another. The high case fatality rates in some regions, especially in non-endemic areas, associated with increasing trend in Brazil, can be explained by the introduction of VL into new geographic areas, accessibility to health care services, delay of diagnosis, timely treatment, clinical management of patients, drug toxicity, comorbidities and host factors (extremes of age, malnutrition and immunosuppression) [3], [14], [15], [32]. It has been shown that the recent introduction of VL into non-endemic areas caused an initial increase of case fatality, probably due to incorrect and/or late diagnosis and medical staff not experienced with this condition [14], [30]. In endemic areas, increased levels of mortality may be a result of occurrence of VL in vulnerable populations, such as those infected with human immunodeficiency virus (HIV) [3], [14]. In fact, VL-HIV/AIDS co-infection is an emerging problem that requires urgent attention in Brazil [15], [35], [36]. Recent changes in the epidemiological profiles of HIV/AIDS and VL, such as the ruralization of HIV and urbanization of VL, indicate more and more geographical overlapping of the transmission areas [35], [36]. HIV serologic tests for patients with VL are of crucial importance, aiming at early diagnosis of co-infection and the reduction of case fatality [35], [37].

The identification of high-risk areas through combination of different spatial analysis techniques may help to define priority areas for specific interventions [38], [39], [40], [41] As spatial distribution of VL in Brazil is heterogeneous, targeted interventions tend to be the most effective control measures [39]. High mortality clusters were identified encompassing areas in all states of the Northeast region, Pará and Tocantins in the North region, several states in the Central-West and Minas Gerais in the Southeast region. We observed a geographical overlap of clusters of municipalities with high mortality rates in endemic areas with active transmission of the disease, according to risk stratification performed by the Brazilian Ministry of Health [42]. Priority to surveillance and control measures is given to municipalities with moderate and intense VL transmission [10], [42]. The VLCSP is in effect since 1985 [2], with control strategies based on detection and treatment of human cases, control of domestic reservoirs and vector control [4], [43]. However, after years of investment, neither incidence nor mortality rates have been reduced significantly and measures did not reduce disease transmission [6], [13], [43], [44].

The wide geographical spread of VL in Brazil is reflected by the emergence of new foci and persistence of old areas of disease occurrence [12], [38]. This may explain why effectiveness of current control measures is suboptimal, to control VL in endemic areas, and to prevent activation or reactivation of foci in areas considered non-endemic [11], [43]. In addition, the domestic dog is a main reservoir, and while accompanying humans in their internal movements through new territories, contributes to disseminate the infection [45]. The emergence of human cases is usually preceded by canine cases [6], [16]. Canine VL is widespread, with up to 20% of dogs infected in localities of high endemicity [44]. Effectiveness of euthanasia of infected dogs for the control of VL transmission is a subject of intense debate [6], [43], [44].

Another factor hampering control is the characteristic of vector *L. longipalpis* to easily adapt to peridomestic areas (such as gardens, parks, and yards) and different temperatures. The vector can be found inside of homes and domestic animal shelters [6], [39], [46]. The geographical distribution of *L. longipalpis* is widespread in Brazil and seems to be expanding [10], [13]. The introduction and spread of VL in capital cities configures an epidemiological reality different from that previously known, requiring a new evidence-based approach for the surveillance and control [6], [13].

Health care and drugs for VL are provided free of charge by a network of public services in Brazil [2], [10], [43]. Although the disease has been known for a long time, the therapeutic arsenal for treatment of leishmaniasis is limited in all endemic countries [47]. The VLCSP has three drugs options available (pentavalent antimonial [first choice], amphotericin B deoxycholate and liposomal amphotericin B) [6], [31], [47]. In addition, these medications are administered through parenteral route, and adverse events are common and may cause kidney damage, pancreatitis and cardiac lesions. Increasing drug resistance has also been observed [47].

Consequently, there are still many challenges for the control of mortality and reduction of case fatality related to VL in Brazil [13]. Further studies are needed for the development of new drugs, therapeutic regimens and clinical management protocols. Control measures should be supported by evidence based on solid methodological grounds. Emphasis should also be given to new diagnostic tests and vaccines. Investment in health education of the population and continuing education programs for health professionals who work in the affected areas is extremely important for early detection and treatment of cases [14]. Transmission risks need to be reduced by control of reservoirs and vectors [5]. The VLCSP should incorporate non-endemic areas in surveillance, aiming at preventing or minimizing spread of the disease [48]. Operational issues in the implementation of preventive measures should also be addressed [13]. These may include deficiencies in the primary health care implementation, with regard to diagnosis, treatment and reporting of VL, evidencing a greater need for integration among control actions and health care for these patients [30].

Our study is subject to limitations. Secondary data may present inconsistencies in the quantity and quality of the information [18]. Despite significant progress in the past years both in coverage and quality of information from *SIM* and *SINAN* databases [12], [18], the number of cases and deaths related to VL may have been underestimated [12]. We aimed to reduce this source of bias by analyzing multiple causes of death instead of merely the underlying causes. The number of deaths could be higher, since 17.3% of deaths due to leishmaniasis (ICD-10: B55) were recorded as unspecified leishmaniasis (ICD-10: B55.9) (799/4,610). This points to deficiencies in the quality of completion of death certificates and the establishment of the underlying cause [12]. A study has shown that after correction of deaths from unspecified leishmaniasis, 10.9% of these were caused by VL as the underlying cause [12].

Other limitations relate to the use of VL cases derived from *SINAN* for the calculation of case fatality rates. Significant underreporting of VL cases occurs in some areas of the country due to deficiencies in access to health services and quality of care and, not apparent clinical manifestation in most cases with *L. infantum* [5]. Since we could not perform a probabilistic linkage among databases, it is unknown whether the same VL cases and deaths are recorded in these databases. Some socio-demographic variables such as race/color, education and marital status presented a considerable proportion of blank data/ignored, which limits the validity and reliability of this result. Despite these limitations, we consider that the results of this study show high representativeness, since all death certificates in a country of continental dimensions were included for a period of more than ten years.

We conclude that VL is an increasing public health problem in Brazil, with high case fatality rates, wide geographic distribution, considerable regional differences, and with a tendency to spread to non-endemic areas. Increasing mortality and case fatality rates in some regions are a cause for concern and require evidence-based response from the health sector. Early diagnosis and institution of effective therapeutic measures are fundamental strategies to reduce case fatality of patients with VL. Our study also shows that spatial analysis for the definition of priority areas provides strong evidence for planning and monitoring surveillance and control of VL.
